# Supplementary material for: Ornaments are equally informative in male and female birds
Source: Nat Commun. 2022 Oct 7;13:5917. doi: 10.1038/s41467-022-33548-7 (PMC9546859; doi:10.1038/s41467-022-33548-7)
Supplement: Supplementary file 1 — Supplementary Information [file 41467_2022_33548_MOESM1_ESM.pdf]

Supplementary Information

Ornaments are equally informative in male and female birds

Sergio Nolazco, Kaspar Delhey, Shinichi Nakagawa, Anne Peters

Contents

- Supplementary Figures (1-3)
- Supplementary Tables (1-5)
- Supplementary References

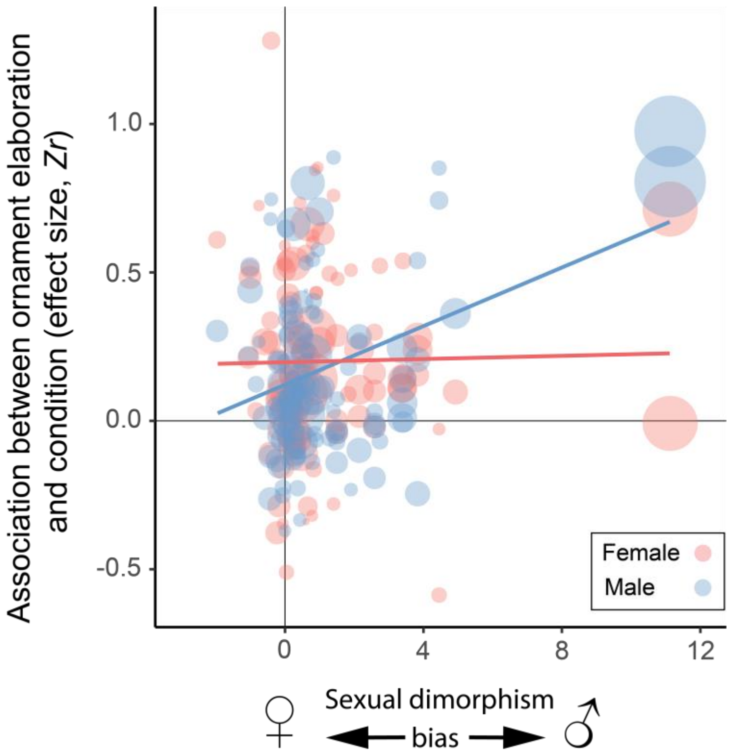

Figure 1. Sexual dimorphism seems to affect condition-dependence of ornaments. Shown are the effect sizes of ornament elaboration and body condition per sex (female: red, male: blue). However, a significant effect of sexual dimorphism was only influenced by four outliers corresponding to a relatively extreme case of sexual dimorphism in the Willow Ptarmigan *Lagopus lagopus* (more details in Results). The size of the bubbles is proportional to the inverse of the standard errors (often referred to as precision), thus the larger the bubble the greater the precision of the effect size. Regression lines are plotted by sex based on the posteriori distributions of the model (details in Methods and Table S1.8). Source data are provided as a Source Data file.

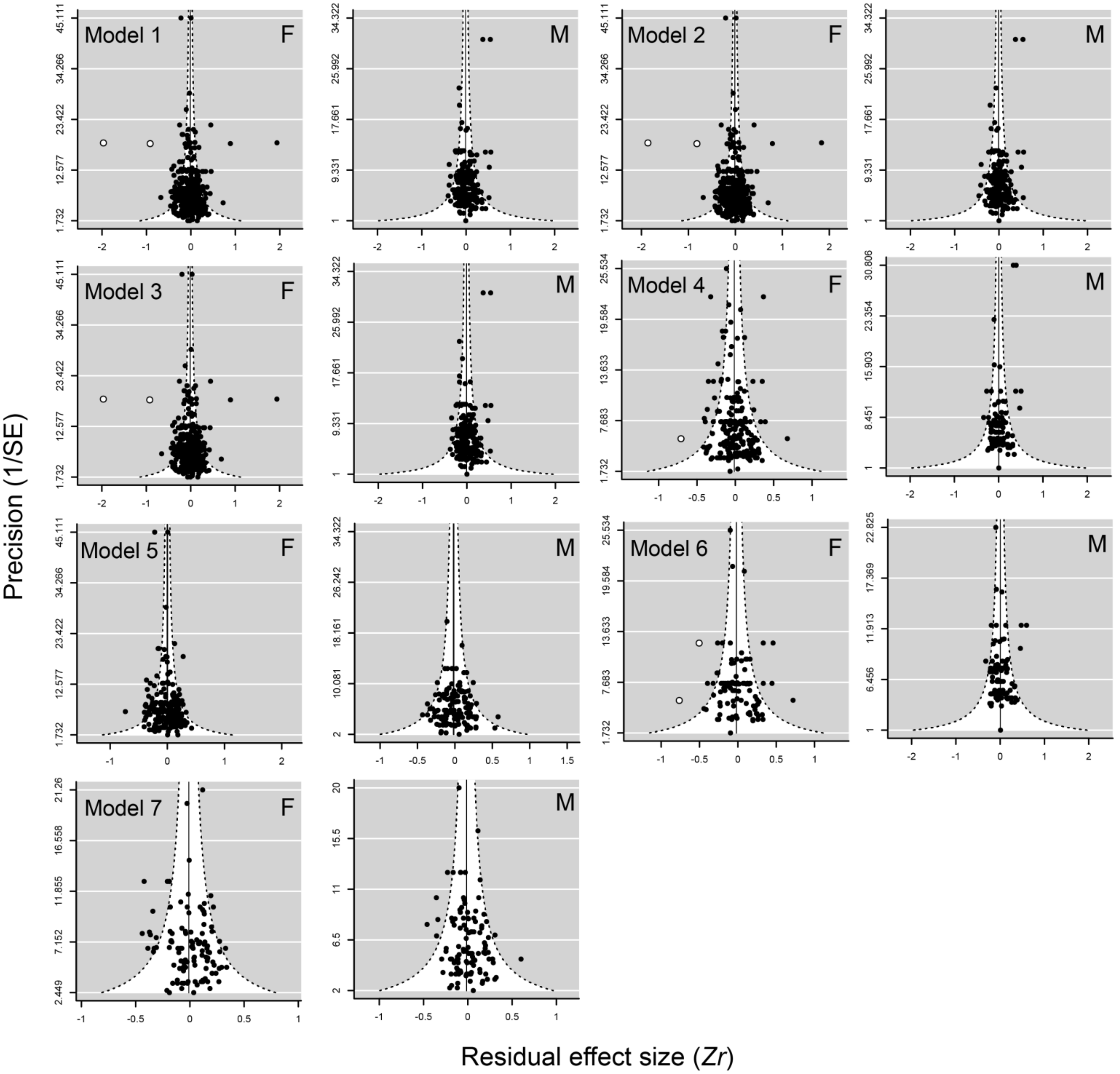

Figure 2. Funnel plots for each main model data set presented by sex (F: females, M: males). Model numeration according to Table S1. Residuals are plotted against the inverse of their standard errors. Each filled dot represents actual residuals, while the empty circles are fabricated residuals using the trim-and-fill method to force funnel plots to be symmetric. The vertical line represents the estimate based on the model along with 95% pseudo confidence intervals (± 1.96xSE) defined by dotted lines. In the absence of publication bias, residuals are expected to lie within this funnel and be evenly distributed on both sides of the vertical line. Deviations from the symmetric funnel shape may indicate publication bias. Results from Egger's tests to test for funnel asymmetry are shown in Table S5. Source data are provided as a Source Data file.

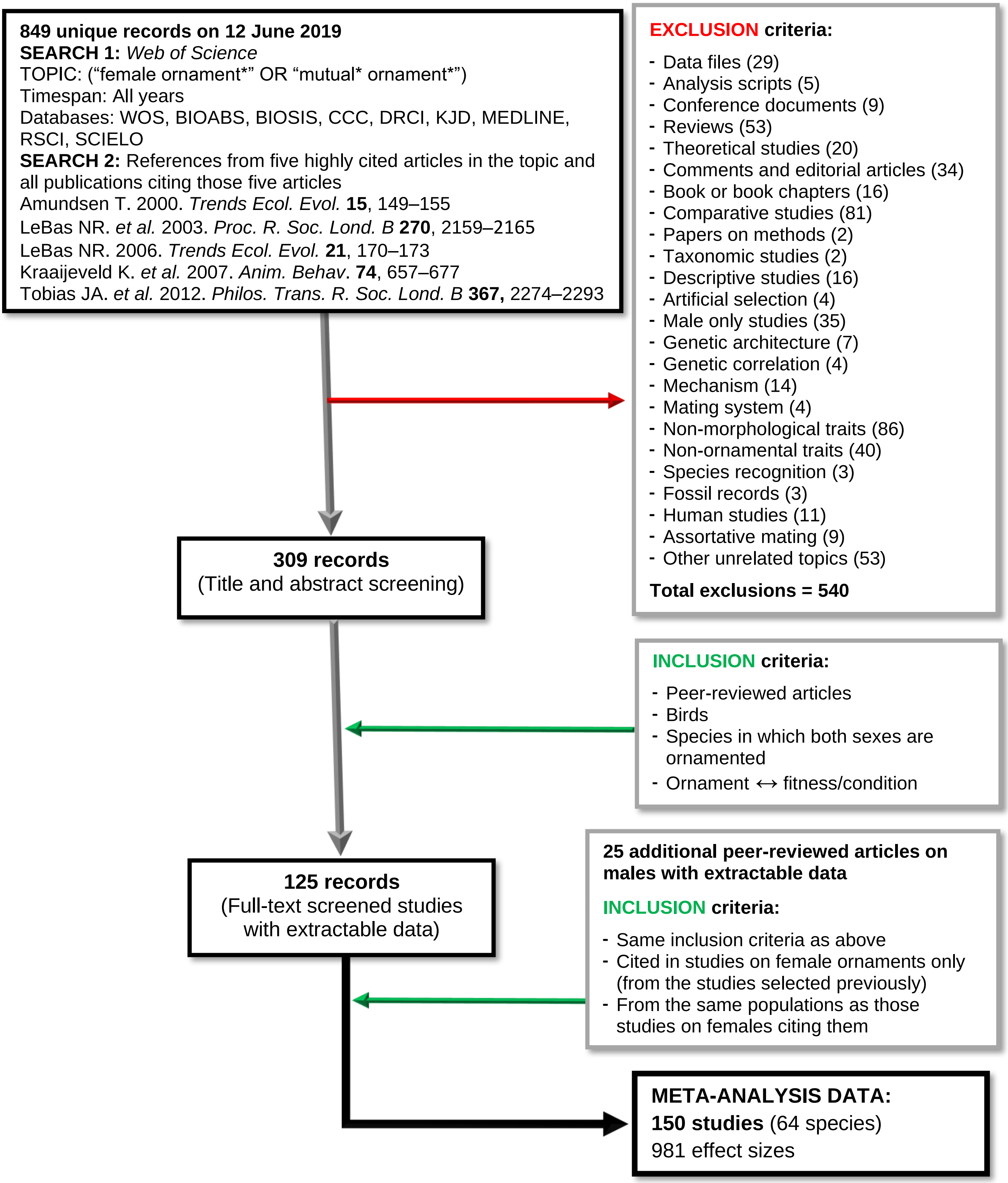

Figure 3. PRISMA flowchart of the literature search strategy and study selection process. Source data are provided as a Source Data file.

Table 1. Summary of meta-analysis model parameters along with 95% credible intervals. Reference level in models 2 to 8 for interactions with Sex is female, in model 2 for Ornament type is carotenoid, in model 3 for Fitness is condition, in model 4 for Condition parameters is body condition, and in model 5 for Fitness parameters is timing of breeding.

Model	Parameter	Posterior mean	Lower 95% CI	Upper 95%CI	Effective sample size
**1: Sex** (Condition & Fitness combined)	Female	0.1944	0.1188	0.2845	3837
	Male	0.2236	0.1159	0.3389	5000
	Publication year (centred mean)	-0.0061	-0.0089	-0.0025	5000
	Phylogeny variance (female)	0.0061	1.52×10^{-11}	0.0166	4866
	Phylogeny variance (male)	0.0137	3.42×10^{-7}	0.0301	4153
	Phylogeny covariance (fem/male)	0.0044	-0.0024	0.0143	4688
	Species ID variance (female)	0.0041	1.46×10^{-11}	0.0112	5000
	Species ID variance (male)	0.0024	2.11×10^{-11}	0.0090	4308
	Species ID covariance (fem/male)	0.0007	-0.0020	0.0048	5000
	Residual variance (female)	0.0658	0.0550	0.0771	5000
	Residual variance (male)	0.0532	0.0425	0.0637	5000
	Residual covariance (fem/male)	0.0150	0.0045	0.0260	5000
**2: Sex*Ornament type** (Condition & Fitness combined)	Female	0.1646	0.0767	0.2551	4796
	Male	0.1402	0.0421	0.2459	4587
	Melanin	-0.0060	-0.1021	0.0821	4619
	Structural	-0.0932	-0.2054	0.0079	5000
	Unpigmented	0.0083	-0.1015	0.1292	5000
	Morphological	0.1482	0.0324	0.2647	5000
	Other	0.0304	-0.0950	0.1511	5000
	Sex:Melanin	0.1337	0.0097	0.2578	5000
	Sex:Structural	0.0501	-0.0891	0.1909	5000
	Sex:Unpigmented	0.0359	-0.1243	0.1941	5000
	Sex:Morphological	0.0065	-0.1451	0.1550	5000
	Sex:Other	0.0644	-0.0904	0.2159	5000
	Publication year (centred mean)	-0.0049	-0.0081	-0.0013	5000
	Phylogeny variance (female)	0.0042	2.39×10^{-13}	0.0153	5000
	Phylogeny variance (male)	0.0071	4.06×10^{-9}	0.0204	5000
	Phylogeny covariance (fem/male)	0.0006	-0.0047	0.0064	4941
	Species ID variance (female)	0.0060	1.21×10^{-10}	0.0157	4751
	Species ID variance (male)	0.0022	4.99×10^{-10}	0.0077	5000
	Species ID covariance (fem/male)	0.0001	-0.0030	0.0037	5000
	Residual variance (female)	0.0640	0.0535	0.0758	4783
	Residual variance (male)	0.0523	0.0425	0.0633	5000
	Residual covariance (fem/male)	0.0137	0.0037	0.0247	4894
**3: Sex*Condition or Fitness**	Female	0.2271	0.1498	0.3020	5000
	Male	0.2609	0.1507	0.3756	4952
	Fitness	-0.0823	-0.1419	-0.0234	5000
	Sex:Fitness	-0.0140	-0.1048	0.0630	5270
	Publication year (centred mean)	-0.0068	-0.0100	-0.0032	5000
	Phylogeny variance (female)	0.0044	7.51×10^{-9}	0.0134	5000
	Phylogeny variance (male)	0.0126	1.18×10^{-9}	0.0293	3440

Model	Parameter	Posterior mean	Lower 95% CI	Upper 95%CI	Effective sample size
	Phylogeny covariance (fem/male)	0.0027	-0.0034	0.0110	3549
	Species ID variance (female)	0.0043	6.74×10^{-10}	0.0108	5000
	Species ID variance (male)	0.0029	2.68×10^{-11}	0.0106	5000
	Species ID covariance (fem/male)	0.0007	-0.0021	0.0049	3710
	Residual variance (female)	0.0650	0.0541	0.0768	5209
	Residual variance (male)	0.0522	0.0420	0.0631	5000
	Residual covariance (fem/male)	0.0140	0.0036	0.0245	5227
**4: Sex*Condition parameters**	Female	0.1770	0.0819	0.2661	5000
	Male	0.2414	0.0834	0.4074	3925
	Body size	0.0481	-0.0568	0.1507	5000
	Immunity	0.0091	-0.1437	0.1578	5000
	Stress	-0.0183	-0.1620	0.1222	5000
	Environment	0.1279	-0.0586	0.3156	5000
	Parasites	0.1182	-0.0898	0.3205	5000
	Sex:Body size	-0.0912	-0.2493	0.0601	5000
	Sex:Immunity	-0.0278	-0.2405	0.1898	5000
	Sex:Stress	0.1499	-0.0720	0.3675	5000
	Sex:Environment	-0.0510	-0.3097	0.1796	5000
	Sex:Parasites	-0.1466	-0.4366	0.1422	5000
	Publication year (centred mean)	-0.0039	-0.0108	0.0030	5263
	Phylogeny variance (female)	0.0037	2.75×10^{-10}	0.0142	5000
	Phylogeny variance (male)	0.0204	6.81×10^{-9}	0.0624	5000
	Phylogeny covariance (fem/male)	0.0020	-0.0065	0.0137	3859
	Species ID variance (female)	0.0051	5.58×10^{-10}	0.0165	5000
	Species ID variance (male)	0.0125	1.24×10^{-9}	0.0346	5000
	Species ID covariance (fem/male)	0.0026	-0.0040	0.0128	5000
	Residual variance (female)	0.0620	0.0452	0.0790	5281
	Residual variance (male)	0.0539	0.0390	0.0680	5571
	Residual covariance (fem/male)	0.0031	-0.0084	0.0156	5000
**5: Sex*Fitness parameters**	Female	0.1528	0.0176	0.3011	5000
	Male	0.1815	0.0446	0.3208	5000
	Reproductive success	0.0347	-0.0875	0.1496	4771
	Offspring quality/condition	0.0668	-0.0718	0.2145	5000
	Parental quality	-0.0798	-0.2295	0.0629	4770
	Survival	-0.1469	-0.3018	0.0286	5000
	Sex:Reproductive success	-0.0383	-0.2026	0.1212	4266
	Sex:Offspring quality/condition	-0.0898	-0.2841	0.1041	4377
	Sex:Parental quality	-0.0074	-0.2106	0.1987	4328
	Sex:Survival	0.0172	-0.2005	0.2485	5000
	Publication year (centred mean)	-0.0082	-0.0123	-0.0044	5000
	Phylogeny variance (female)	0.0078	1.84×10^{-10}	0.0263	5000
	Phylogeny variance (male)	0.0058	2.00×10^{-9}	0.0192	5000
	Phylogeny covariance (fem/male)	0.0013	-0.0044	0.0092	5000
	Species ID variance (female)	0.0064	1.81×10^{-10}	0.0176	5000
	Species ID variance (male)	0.0020	1.01×10^{-11}	0.0076	5324
	Species ID covariance (fem/male)	0.0001	-0.0031	0.0038	5000

Model	Parameter	Posterior mean	Lower 95% CI	Upper 95%CI	Effective sample size
	Residual variance (female)	0.0760	0.0598	0.0923	5000
	Residual variance (male)	0.0661	0.0498	0.0835	5000
	Residual covariance (fem/male)	0.0172	$6.59x10^{-6}$	0.0346	4765
**6: Sex*Sexual dimorphism** (Condition data only) Removing outliers	Female	0.2009	0.0861	0.3215	5000
	Male	0.1450	0.0206	0.2794	5000
	Dimorphism	-0.0121	-0.0733	0.0496	5000
	Sex:Dimorphism	0.0282	-0.0502	0.1038	5000
	Publication year (centred mean)	-0.0108	-0.0220	0.0005	5000
	Phylogeny variance (female)	0.0072	$2.11x10^{-10}$	0.0289	4544
	Phylogeny variance (male)	0.0103	$1.45x10^{-10}$	0.0359	5000
	Phylogeny covariance (fem/male)	0.0017	-0.0063	0.0133	5000
	Species ID variance (female)	0.0053	$3.70x10^{-11}$	0.0210	5000
	Species ID variance (male)	0.0070	$1.71x10^{-14}$	0.0236	5000
	Species ID covariance (fem/male)	0.0009	-0.0045	0.0087	5000
	Residual variance (female)	0.0848	0.0574	0.1150	5000
	Residual variance (male)	0.0705	0.0476	0.0948	4455
	Residual covariance (fem/male)	0.0072	-0.0118	0.0279	5205
**7: Sex*Sexual dimorphism** (Fitness data only)	Female	0.1317	0.0061	0.2593	5000
	Male	0.1466	0.0083	0.2833	5000
	Dimorphism	0.0015	-0.0558	0.0613	5283
	Sex:Dimorphism	-0.0230	-0.0954	0.0523	5000
	Publication year (centred mean)	-0.0087	-0.0178	0.0001	5000
	Phylogeny variance (female)	0.0078	$1.22x10^{-9}$	0.0287	5000
	Phylogeny variance (male)	0.0088	$9.28x10^{-11}$	0.0332	5212
	Phylogeny covariance (fem/male)	0.0012	-0.0075	0.0109	5144
	Species ID variance (female)	0.0036	$3.78x10^{-11}$	0.0141	4760
	Species ID variance (male)	0.0046	$6.14x10^{-12}$	0.0169	5071
	Species ID covariance (fem/male)	0.0002	-0.0041	0.0050	5000
	Residual variance (female)	0.0720	0.0497	0.0962	5000
	Residual variance (male)	0.0728	0.0484	0.0988	5000
	Residual covariance (fem/male)	0.0198	0.0022	0.0385	5000
**8: Sex*Sexual dimorphism** (Condition data only) Without removing outliers	Female	0.1948	0.0808	0.3127	5000
	Male	0.1333	0.0075	0.2756	4617
	Dimorphism	0.0040	-0.0308	0.0387	4762
	Sex:Dimorphism	0.0451	0.0013	0.0901	5000
	Publication year (centred mean)	-0.0118	-0.0241	-0.0009	4436
	Phylogeny variance (female)	0.0070	0.0000	0.0276	5000
	Phylogeny variance (male)	0.0120	0.0000	0.0417	5000
	Phylogeny covariance (fem/male)	0.0022	-0.0065	0.0152	5000
	Species ID variance (female)	0.0052	0.0000	0.0205	5000
	Species ID variance (male)	0.0080	0.0000	0.0262	5000
	Species ID covariance (fem/male)	0.0013	-0.0046	0.0101	5000
	Residual variance (female)	0.0871	0.0572	0.1164	5000
	Residual variance (male)	0.0697	0.0481	0.0958	5000
	Residual covariance (fem/male)	0.0084	-0.0101	0.0279	5000

Table 2. Summary of an additional general model testing the effect of Study type (correlational or experimental). Reference level is correlational.

Model	Parameter	Posterior mean	Lower 95% CI	Upper 95%CI	Effective sample size
**Sex + Study type** (Condition & Fitness combined)	Female	0.193	0.116	0.277	5000
	Male	0.223	0.115	0.335	4679
	Study type (experimental)	0.016	-0.081	0.115	5000
	Publication year (centred mean)	-0.006	-0.010	-0.003	3615
	Phylogeny variance (female)	0.006	$7.4\text{x}10^{-11}$	0.016	5000
	Phylogeny variance (male)	0.014	$9.8\text{x}10^{-8}$	0.030	4586
	Phylogeny covariance (fem/male)	0.004	-0.003	0.013	3615
	Species ID variance (female)	0.004	$1.3\text{x}10^{-10}$	0.011	5000
	Species ID variance (male)	0.002	$3.0\text{x}10^{-11}$	0.009	4959
	Species ID covariance (fem/male)	0.001	-0.002	0.005	4450
	Residual variance (female)	0.066	0.055	0.077	4748
	Residual variance (male)	0.053	0.043	0.064	5000
	Residual covariance (fem/male)	0.015	0.004	0.026	5000

Table 3. Summary of parameters along with 95% credible intervals used to construct Figure 2. Mean sex differences are also presented along with 95% CIs.

Model	Parameter	Sex (*n*)	Estimate	Lower 95% CI	Upper 95%CI
**1: Sex** (Condition & Fitness combined)	Sex only	F (510)	0.194	0.119	0.284
		M (471)	0.224	0.116	0.339
		ΔF-M	-0.029	-0.142	0.079
**2. Sex*Ornament type** (Condition & Fitness combined)	Carotenoid	F (145)	0.165	0.077	0.255
		M (140)	0.140	0.042	0.246
		ΔF-M	0.024	-0.097	0.155
	Melanin	F (103)	0.159	0.058	0.251
		M (85)	0.268	0.166	0.384
		ΔF-M	-0.109	-0.246	0.036
	Structural	F (77)	0.071	-0.042	0.180
		M (71)	0.097	-0.013	0.226
		ΔF-M	-0.026	-0.179	0.128
	Unpigmented	F (71)	0.173	0.053	0.291
		M (47)	0.184	0.047	0.328
		ΔF-M	-0.011	-0.178	0.177
	Morphological	F (55)	0.313	0.208	0.419
		M (74)	0.295	0.189	0.408
		ΔF-M	0.018	-0.118	0.167
	Other	F (59)	0.195	0.071	0.316
		M (54)	0.235	0.114	0.361
		ΔF-M	-0.040	-0.205	0.120
**3: Sex*Condition or Fitness** (Condition & Fitness separated)	Condition	F (221)	0.227	0.149	0.302
		M (200)	0.261	0.151	0.376
		ΔF-M	-0.034	-0.157	0.081
	Fitness	F (289)	0.145	0.068	0.226
		M (271)	0.164	0.054	0.284
		ΔF-M	-0.020	-0.144	0.101
**4: Sex*Condition parameters**	Body condition	F (74)	0.177	0.082	0.266
		M (72)	0.241	0.083	0.407
		ΔF-M	-0.064	-0.243	0.105
	Body size	F (63)	0.225	0.131	0.330
		M (51)	0.198	0.024	0.364
		ΔF-M	0.027	-0.169	0.203
	Immunity	F (30)	0.186	0.042	0.325
		M (28)	0.223	0.037	0.418
		ΔF-M	-0.037	-0.256	0.197
	Stress	F (33)	0.159	0.023	0.296
		M (19)	0.373	0.172	0.588
		ΔF-M	-0.214	-0.460	0.020
	Environment	F (11)	0.305	0.128	0.481
		M (21)	0.318	0.113	0.517
		ΔF-M	-0.013	-0.282	0.231
	Parasites	F (10)	0.295	0.087	0.483
		M (9)	0.213	-0.022	0.453
		ΔF-M	0.082	-0.199	0.390

**Model**	**Parameter**	**Sex (*n*)**	**Estimate**	**Lower 95% CI**	**Upper 95%CI**
		F (126)	0.187	0.070	0.305
	Reproductive success	M (127)	0.178	0.069	0.290
		ΔF-M	0.009	-0.139	0.163
		F (55)	0.220	0.087	0.358
	Offspring quality/condition	M (49)	0.158	0.024	0.292
		ΔF-M	0.061	-0.117	0.248
		F (44)	0.073	-0.066	0.237
**5: Sex*Fitness parameters**	Parental quality	M (43)	0.094	-0.045	0.256
		ΔF-M	-0.021	-0.232	0.177
		F (40)	0.153	0.018	0.301
	Timing of breeding	M (34)	0.181	0.045	0.321
		ΔF-M	-0.029	-0.219	0.145
		F (24)	0.006	-0.155	0.174
	Survival	M (18)	0.052	-0.117	0.213
		ΔF-M	-0.046	-0.256	0.176

Table 4. Summary of variances per sex transformed into proportions and covariances transformed into correlation coefficients, along with 95% credible intervals.

Model	Parameter	Variance	Lower 95% CI	Upper 95%CI	Posterior mean (*r*)	Lower 95% CI	Upper 95%CI
1: Sex<br>(Condition & Fitness combined)	Phylogeny	F:0.08<br>M:0.19	2.3×10^{-10}<br>5.3×10^{-6}	0.20<br>0.36	0.5061	-0.2172	0.9989
	Species ID	F:0.05<br>M:0.03	1.7×10^{-10}<br>2.9×10^{-10}	0.14<br>0.13	0.1772	-0.7150	0.9613
	Residual	F:0.87<br>M:0.78	0.74<br>0.60	0.98<br>0.94	0.2538	0.0861	0.4281
2: Sex*Ornament type<br>(Condition & Fitness combined)	Phylogeny	F:0.05<br>M:0.11	4.0×10^{-12}<br>6.7×10^{-8}	0.19<br>0.28	0.1401	-0.6952	0.9729
	Species ID	F:0.08<br>M:0.04	1.6×10^{-9}<br>8.9×10^{-9}	0.20<br>0.12	0.0318	-0.8515	0.8563
	Residual	F:0.87<br>M:0.85	0.72<br>0.69	0.99<br>0.99	0.2373	0.0636	0.4114
3: Sex*Condition or Fitness<br>(Condition & Fitness separated)	Phylogeny	F:0.06<br>M:0.18	1.0×10^{-7}<br>1.7×10^{-8}	0.17<br>0.36	0.3729	-0.4620	0.9897
	Species ID	F:0.06<br>M:0.04	9.3×10^{-9}<br>3.4×10^{-10}	0.14<br>0.16	0.1683	-0.7234	0.9380
	Residual	F:0.88<br>M:0.78	0.77<br>0.60	0.99<br>0.94	0.2404	0.0656	0.4090
4: Sex*Condition parameters	Phylogeny	F:0.05<br>M:0.21	4.1×10^{-9}<br>9.9×10^{-8}	0.18<br>0.53	0.1740	-0.7366	0.9719
	Species ID	F:0.07<br>M:0.15	8.3×10^{-9}<br>1.3×10^{-8}	0.21<br>0.38	0.2751	-0.6381	0.9865
	Residual	F:0.88<br>M:0.64	0.71<br>0.39	0.99<br>0.88	0.0543	-0.1553	0.2532
5: Sex*Fitness parameters	Phylogeny	F:0.08<br>M:0.07	2.5×10^{-9}<br>3.2×10^{-8}	0.25<br>0.23	0.2019	-0.7030	0.9610
	Species ID	F:0.07<br>M:0.02	2.5×10^{-9}<br>1.6×10^{-10}	0.18<br>0.10	0.0400	-0.8571	0.8849
	Residual	F:0.85<br>M:0.90	0.69<br>0.74	0.99<br>0.99	0.2416	0.0240	0.4781
6: Sex*Sexual dimorphism<br>(Condition data only)<br>Removing outliers	Phylogeny	F:0.07<br>M:0.11	2.6×10^{-9}<br>2.2×10^{-9}	0.25<br>0.33	0.1669	-0.7059	0.9923
	Species ID	F:0.05<br>M:0.08	3.4×10^{-10}<br>1.7×10^{-13}	0.19<br>0.24	0.1203	-0.7778	0.9526
	Residual	F:0.88<br>M:0.82	0.67<br>0.59	0.99<br>0.99	0.0926	-0.1444	0.3469
7: Sex*Sexual dimorphism<br>(Fitness data only)	Phylogeny	F:0.08<br>M:0.09	1.3×10^{-8}<br>1.1×10^{-9}	0.29<br>0.31	0.1231	-0.7322	0.9732
	Species ID	F:0.04<br>M:0.05	4.9×10^{-10}<br>8.3×10^{-11}	0.15<br>0.18	0.0260	-0.8260	0.9226
	Residual	F:0.88<br>M:0.86	0.65<br>0.63	0.99<br>0.99	0.2715	0.0513	0.4868
8: Sex*Sexual dimorphism<br>(Condition data only)<br>Without removing outliers	Phylogeny	F:0.06<br>M:0.12	2.3×10^{-9}<br>1.6×10^{-9}	0.23<br>0.37	0.1903	-0.7079	0.9798
	Species ID	F:0.05<br>M:0.09	1.8×10^{-9}<br>2.6×10^{-8}	0.18<br>0.27	0.1612	-0.7404	0.9539
	Residual	F:0.89<br>M:0.79	0.68<br>0.56	0.99<br>0.99	0.1073	-0.1336	0.3316

Table 5. Publication bias does occur in data for both sexes, however results for Egger's tests and trim-and-fill analysis suggest that publication bias is relatively small across models, with very few missing samples (*k*) identified and negligible changes in effect size after correction.

		Egger's test			Trim-and-fill method		
**Model**	**Sex**	***t***	***df***	***P***	***k***	***Zr***	**95% CI**
1: Sex (Condition & Fitness combined)	F	0.359	508	0.720	-2	-0.012	-0.035 – 0.013
	M	-2.839	469	0.005	0	-0.012	-0.030 – 0.006
2: Sex*Ornament type (Condition & Fitness combined)	F	0.466	508	0.641	-2	-0.011	-0.033 – 0.012
	M	-3.083	469	0.002	0	-0.012	-0.029 – 0.006
3: Sex*Condition or Fitness (Condition & Fitness separated)	F	-0.109	508	0.913	-2	-0.011	-0.034 – 0.012
	M	-3.182	469	0.002	0	-0.009	-0.027 – 0.008
4: Sex*Condition parameters	F	1.941	219	0.053	-1	-0.015	-0.040 – 0.010
	M	-5.272	198	<0.0001	0	0.0002	-0.026 – 0.027
5: Sex*Fitness parameters	F	-0.077	287	0.939	0	0.0003	-0.029 – 0.030
	M	0.334	269	0.739	0	-0.016	-0.038 – 0.005
6: Sex*Sexual Dimorphism (Condition data only)	F	0.735	103	0.464	-2	-0.020	-0.062 – 0.021
	M	-0.107	103	0.915	0	0.002	-0.035 – 0.040
7: Sex*Sexual Dimorphism (Fitness data only)	F	0.974	110	0.332	0	-0.011	-0.048 – 0.026
	M	1.816	110	0.072	0	-0.015	-0.048 – 0.019

Egger's (two-sided) test results are shown for sex-specific data sets per model (*t* values, degrees of freedom and *P* values), estimated number of missing samples with the sign indicating the direction of bias correction (*k*), mean effect size estimate after correction (*Zr*) together with its 95% credible interval.

Supplementary References (for Figure 1 in the main text)

[1] Quinard, A., Cézilly, F., Motreuil, S., Rossi, J-M. & Biard, C. Reduced sexual dichromatism, mutual ornamentation, and individual quality in the monogamous zenaida dove, *Zenaida aurita*. *J. Avian Biol.* **48**, 489–501 (2017).

[2] Martínez, J. E., Calvo, J. F., Jiménez-Franco, M. V., Zuberogoitia, I. & López-López, P. Colour morph does not predict brood size in the booted eagle. *Ornis Fenn.* **93**, 130–136 (2016).

[3] Penteriani, V., Alonso-Alvarez, C., del Mar Delgado, M., Sergio, F. & Ferrer M. Brightness variability in the white badge of the eagle owl *Bubo bubo*. *J. Avian Biol.* **37**, 110–116 (2006).

[4] Avilés, J. M. & Parejo, D. Covariation between bill colouration and fitness components in a nocturnal bird. *J. Avian Biol.* **43**, 565–570 (2012).

[5] Roulin, A., Richner, H. & Ducrest, A-L. Genetic, environmental, and condition-dependent effects on female and male ornamentation in the barn owl *Tyto alba*. *Evolution* **52**, 1451–1460 (1998).

[6] Roulin, A., Jungi, T. W., Pfister, H. & Dijkstra, C. Female barn owls (*Tyto alba*) advertise good genes. *Proc. R. Soc. Lond. B* **267**, 937–941 (2000).

[7] Roulin, A., Dijkstra, C., Riols, C. & Ducrest, A-L. Female- and male-specific signals of quality in the barn owl. *J. Evol. Biol.* **14**, 255–266 (2001a).

[8] Roulin, A., Riols, C., Dijkstra, C. & Ducrest, A-L. Female plumage spottiness signals parasite resistance in the barn owl (*Tyto alba*). *Behav. Ecol.* **12**, 103–110 (2001b).

[9] Roulin, A., Ducrest, A-L., Balloux, F., Dijkstra, C. & Riols, C. A female melanin ornament signals offspring fluctuating asymmetry in the barn owl. *Proc. R. Soc. Lond. B* **270**, 167–171 (2003).

[10] Roulin, A. Proximate basis of the covariation between a melanin-based female ornament and offspring quality. *Oecologia* **140**, 668–675 (2004).

[11] Roulin, A. Covariation between eumelanic pigmentation and body mass only under specific conditions. *Naturwissenschaften* **96**, 375–382 (2009).

[12] Dreiss, A., Henry, I., Ruppli, C., Almasi, B. & Roulin, A. Darker eumelanic barn owls better withstand food depletion through resistance to food deprivation and lower appetite. *Oecologia* **164**, 65–71 (2010).

[13] Almasi, B., Roulin, A., Korner-Nievergelt, F., Jenni-Eiermann, S. & Jenni, L. Coloration signals the ability to cope with elevated stress hormones: effects of corticosterone on growth of barn owls are associated with melanism. *J. Evol. Biol.* **25**, 1189–1199 (2012).

[14] van den Brink, V., Dolivo, V., Falourd, X., Dreiss, A. N. & Roulin, A. Melanic color-dependent antipredator behavior strategies in barn owl nestlings. *Behav. Ecol.* **23**, 473–480 (2012).

[15] Wiebe, K. L. Assortative mating by color in a population of hybrid northern flickers. *Auk* **117**, 525–529 (2000).

[16] Murphy, T. G. Racketed tail of the male and female turquoise-browed motmot: male but not female tail length correlates with pairing success, performance, and reproductive success. *Behav. Ecol. Sociobiol.* **61**, 911–918 (2007).

[17] Murphy, T. G. & Pham, T. T. Condition and brightness of structural blue-green: motmot tail-racket brightness is related to speed of feather growth in males, but not in females. *Biol. J. Linn. Soc.* **106**, 673–681 (2012).

[18] Silva, N., Avilés, J. M., Danchin, E. & Parejo, D. Informative content of multiple plumage-coloured traits in female and male European rollers. *Behav. Ecol. Sociobiol.* **62**, 1969 (2008).

[19] Tella, J. L., Forfero, M. G., Donázar, J. A. & Hiraldo, F. Is the expression of male traits in female lesser kestrels related to sexual selection? *Ethology* **103**, 72–81 (1997).

[20] Vergara, P., Fargallo, J., Martínez-Padilla, J. & Lemus, J. Inter-annual variation and information content of melanin-based coloration in female Eurasian kestrels. *Biol. J. Linn. Soc.* **97**, 781–790 (2009).

[21] Parejo, D., Silva, N., Danchin, É. & Avilés, J. M. Informative content of melanin-based plumage colour in adult Eurasian kestrels. *J. Avian Biol.* **42**, 49–60 (2011).

[22] Vergara, P., Fargallo, J. A. & Martínez-Padilla, J. Genetic basis and fitness correlates of dynamic carotenoid-based ornamental coloration in male and female common kestrels *Falco tinnunculus*. *J. Evol. Biol.* **28**, 146–154 (2015).

[23] López-Idiáquez, D., Vergara, P., Fargallo, J. A. & Martínez-Padilla, J. Old males reduce melanin-

pigmented traits and increase reproductive outcome under worse environmental conditions in common kestrels. *Ecol. Evol.* **6**, 1224–1235 (2016).

[24] Van Rooij, E. P. & Griffith, S. C. No evidence of assortative mating on the basis of putative ornamental traits in long-tailed finches *Poephila acuticauda*. *Ibis* **154**, 444–451 (2012).

[25] Simons, M. J. P. et al. Bill redness is positively associated with reproduction and survival in male and female zebra finches. *PloS One* **7**, e40721 (2012).

[26] Funghi, C., Trigo, S., Gomes, A. C. R., Soares, M. C. & Cardoso, G. C. Release from ecological constraint erases sex difference in social ornamentation. *Behav. Ecol. Sociobiol.* **72**, 67 (2018).

[27] Pilastro, A., Griggio, M. & Matessi, G. Male rock sparrows adjust their breeding strategy according to female ornamentation: parental or mating investment? *Anim. Behav.* **66**, 265–271 (2003).

[28] Griggio, M., Zanollo, V. & Hoi, H. Female ornamentation, parental quality, and competitive ability in the rock sparrow. *J. Ethol.* **28**, 455–462 (2010).

[29] García-Navas, V., García del Rincón, A., Ferrer, E. S. & Fathi, H. Mating strategies, parental investment and mutual ornamentation in Iberian rock sparrows (*Petronia petronia*). *Behaviour* **150**, 1641–1663 (2013).

[30] Cantarero, A., López-Arrabé, J., Palma, A. & Moreno, J. Oxidative status in nestlings shows different associations with parental carotenoid-based plumage ornaments depending on parental sex and year: a study of rock sparrows *Petronia petronia*. *Ethol. Ecol. Evol.* **29**, 521–541 (2017).

[31] Linville, S. U., Breitwisch, R. & Schilling, A. J. Plumage brightness as an indicator of parental care in northern cardinals. *Anim. Behav.* **55**, 119–127 (1998).

[32] Jawor, J. M. & Breitwisch, R. Multiple ornaments in male northern cardinals, *Cardinalis cardinalis*, as indicators of condition. *Ethology* **110**, 113–126 (2004).

[33] Jawor, J. M., Gray, N., Beall, S. M. & Breitwisch, R. Multiple ornaments correlate with aspects of condition and behaviour in female northern cardinals, *Cardinalis cardinalis. Anim. Behav*. **67**, 875–882 (2004).

[34] Grunst, A. S. et al. Disruptive selection on plumage colouration across genetically determined morphs. *Anim. Behav*. **124**, 97–108 (2017).

[35] Grunst, A. S., Rotenberry, J. T. & Grunst, M. L. Age-dependent relationships between multiple sexual pigments and condition in males and females. *Behav. Ecol*. **25**, 276–287 (2014).

[36] Reudink, M. W., Marra, P. P., Boag, P. T. & Ratcliffe, L. M. Plumage coloration predicts paternity and polygyny in the American redstart. *Anim. Behav.* **77**, 495–501 (2009a).

[37] Reudink, M. W., Studds, C. E., Marra, P. P., Kurt Kyser, T. & Ratcliffe, L. M. Plumage brightness predicts non-breeding season territory quality in a long-distance migratory songbird, the American redstart *Setophaga ruticilla*. *J. Avian Biol.* **40**, 34–41 (2009b).

[38] Germain, R. R., Reudink, M. W., Marra, P. P. & Ratcliffe, L. M. Carotenoid-based male plumage predicts parental investment in the American redstart. *Wilson J. Ornithol.* **122**, 318–325 (2010).

[39] Osmond, M. M. et al. Relationships between carotenoid-based female plumage and age, reproduction and mate color in the American redstart (*Setophaga ruticilla*). *Can. J. Zool*. **91**, 589–595 (2013).

[40] Bulluck, L. P. et al. Feather carotenoid content is correlated with reproductive success and provisioning rate in female prothonotary warblers. *Auk* **134**, 229–239 (2017).

[41] Slevin, M. C., Bulluck, L. P., Matthews, A. E. & Boves, T. J. Spatial variation in carotenoid plumage coloration and relationships between female coloration and quality in prothonotary warblers (*Protonotaria citrea*). *Auk* **136**, ukz011 (2019).

[42] Dunn, P. O., Garvin, J. C., Whittingham, L. A., Freeman-Gallant, C. R. & Hasselquist, D. Carotenoid and melanin-based ornaments signal similar aspects of male quality in two populations of the common yellowthroat. *Funct. Ecol.* **24**, 149–158 (2010).

[43] Freeman-Gallant, C. R. et al. Sexual selection, multiple male ornaments, and age- and condition-dependent signaling in the common yellowthroat. *Evolution* **64**, 1007–1017 (2010).

[44] Freeman-Gallant, C. R. et al. Oxidative damage to DNA related to survivorship and carotenoid-based sexual ornamentation in the common yellowthroat. *Biol. Lett*. **7**, 429–432 (2011).

[45] Freeman-Gallant, C. R., Schneider, R. L., Taff, C. C., Dunn, P. O. & Whittingham, L. A. Contrasting patterns of selection on the size and coloration of a

female plumage ornament in common yellowthroats. *J. Evol. Biol.* **27**, 982–991 (2014).

[46] Eckert, C. G. & Weatherhead, P. J. Male characteristics, parental quality and the study of mate choice in the red-winged blackbird (*Agelaius phoeniceus*). *Behav. Ecol. Sociobiol.* **20**, 35–42 (1987).

[47] Muma, K. E. & Weatherhead, P. J. Male traits expressed in females: direct or indirect sexual selection? *Behav. Ecol. Sociobiol.* **25**, 23–31 (1989).

[48] Cabezas, S., Tella, J. L., Carrete, M. & Bortolotti, G. R. Is the rufous axillary patch of the screaming cowbird a secondary sexual character? *Ardeola* **58**, 309–313 (2011).

[49] Rosen, R. F. & Tarvin, K. A. Sexual signals of the male American goldfinch. *Ethology* **112**,1008–1019 (2006).

[50] Kelly, R. J., Murphy, T. G., Tarvin, K. A. & Burness, G. Carotenoid-based ornaments of female and male American goldfinches (*Spinus tristis*) show sex-specific correlations with immune function and metabolic rate. *Physiol. Biochem. Zool.* **85**, 348–363 (2012).

[51] Tarvin, K. A. et al. Dynamic status signal reflects outcome of social interactions, but not energetic stress. *Front. Ecol. Evol.* **4**, 79 (2016).

[52] Hill, G. E. Female house finches prefer colourful males: sexual selection for a condition-dependent trait. *Anim. Behav.* **40**, 563–572 (1990).

[53] Hill, G. E. Plumage coloration is a sexually selected indicator of male quality. *Nature* **350**, 337–339 (1991).

[54] Hill, G. E. Male mate choice and the evolution of female plumage coloration in the house finch. *Evolution* **47**, 1515–1525 (1993).

[55] Amundsen, T., Forsgren, E. & Hansen, L. T. T. On the function of female ornaments: male bluethroats prefer colourful females. *Proc. R. Soc. Lond. B* **264**, 1579-1586 (1997).

[56] Johnsen, A., Andersson, S., Örnborg, J. & Lifjeld, J. T. Ultraviolet plumage ornamentation affects social mate choice and sperm competition in bluethroats (Aves: *Luscinia s. svecica*): a field experiment. *Proc. R. Soc. Lond. B* **265**, 1313–1318 (1998).

[57] Smiseth, P. T. & Amundsen, T. Does female plumage coloration signal parental quality? A male removal experiment with the bluethroat (*Luscinia s. svecica*). *Behav. Ecol. Sociobiol.* **47**, 205–212 (2000).

[58] Johnsen, A., Lifjeld, J., Andersson, S., Örnborg, J. & Amundsen, T. Male characteristics and fertilisation success in bluethroats. *Behaviour* **138**, 1371–1390 (2001).

[59] Pärn, H., Lifjeld, J. T. & Amundsen, T. Female throat ornamentation does not reflect cell-mediated immune response in bluethroats *Luscinia s. svecica*. *Oecologia* **146**, 496–504 (2005).

[60] Potti, J. & Montalvo, S. Male arrival and female mate choice in the pied flycatcher *Ficedula hypoleuca* in central Spain. *Ornis Scand*. **22**, 45–54 (1991).

[61] Potti, J. & Merino, S. Decreased levels of blood trypanosome infection correlate with female expression of a male secondary sexual trait: implications for sexual selection. *Proc. R. Soc. Lond. B* **263**, 1199–1204 (1996).

[62] Gil, D., Lacroix, A. & Potti, J. Within-clutch variation in yolk androgens in relation to female expression of a male ornament in pied flycatchers *Ficedula hypoleuca*. *Ardeola* **53**, 307–315 (2006).

[63] Morales, J. et al. Female ornaments in the pied flycatcher *Ficedula hypoleuca*: associations with age, health and reproductive success. *Ibis* **149**, 245–254 (2007).

[64] Canal, D., Dávila, J. & Potti, J. Male phenotype predicts extra-pair paternity in pied flycatchers. *Behaviour* **148**, 691–712 (2011).

[65] Ruiz-de-Castañeda, R., Burtt, E. H., González-Braojos, S. & Moreno, J. Bacterial degradability of an intrafeather unmelanized ornament: a role for feather-degrading bacteria in sexual selection? *Biol. J. Linn. Soc.* **105**, 409–419 (2012).

[66] Potti, J., Canal, D. & Serrano, D. Lifetime fitness and age-related female ornament signalling: evidence for survival and fecundity selection in the pied flycatcher. *J. Evol. Biol.* **26**, 1445–1457 (2013).

[67] López-Arrabé, J., Cantarero, A., Pérez-Rodríguez, L., Palma, A. & Moreno, J. Plumage ornaments and reproductive investment in relation to oxidative status in the pied flycatcher (*Ficedula hypoleuca iberiae). Can. J. Zool.* **92**, 1019–1027 (2014).

[68] Moreno, J., Cantarero, A., Plaza, M. & Lopez-Arrabé, J. Phenotypic plasticity in breeding plumage signals in both sexes of a migratory bird: responses to breeding conditions. *J. Avian Biol.* **50**, 1–11 (2019).

[69] Török, J., Hegyi, G. & Garamszegi, L. Z. Depigmented wing patch size is a condition-dependent indicator of viability in male collared flycatchers. *Behav. Ecol.* **14**, 382–388 (2003).

[70] Hegyi, G. et al. Phenotypic plasticity in a conspicuous female plumage trait: information content and mating patterns. *Anim. Behav.* **75**, 977–989 (2008).

[71] Kiss, D., Hegyi, G., Török, J. & Rosivall, B. The relationship between maternal ornamentation and feeding rate is explained by intrinsic nestling quality. *Behav. Ecol. Sociobiol.* **67**, 185–192 (2013).

[72] Laczi, M. et al. Integrated plumage colour variation in relation to body condition, reproductive investment and laying date in the collared flycatcher. *Naturwissenschaften* **100**, 983–991 (2013).

[73] Kötél, D., Laczi, M., Török, J. & Hegyi, G. Mutual ornamentation and the parental behaviour of male and female collared flycatchers *Ficedula albicollis* during incubation. *Ibis* **158**, 796–807 (2016).

[74] Laczi, M., Kötél, D., Török, J. & Hegyi, G. Mutual plumage ornamentation and biparental care: consequences for success in different environments. *Behav. Ecol.* **28**, 1359–1368 (2017).

[75] Rowe, K. M. & Weatherhead, P. J. Assortative mating in relation to plumage traits shared by male and female American robins. *Condor* **113**, 881–889 (2011).

[76] Balenger, S. L., Johnson, L. S., Brubaker, J. L. & Ostlind, E. Parental effort in relation to structural plumage coloration in the mountain bluebird (*Sialia currucoides*). *Ethology* **113**, 838–846 (2007).

[77] Morrison, A., Flood, N. J. & Reudink, M. W. Reproductive correlates of plumage coloration of female mountain bluebirds: female mountain bluebird coloration. *J. Field Ornithol.* **85**, 168–179 (2014).

[78] Siefferman, L. & Hill, G. E. Structural and melanin coloration indicate parental effort and reproductive success in male eastern bluebirds. *Behav. Ecol.* **14**, 855–861 (2003).

[79] Siefferman, L. & Hill, G. E. Evidence for sexual selection on structural plumage coloration in female eastern bluebirds (*Sialia sialis*). *Evolution* **59**, 1819–1828 (2005).

[80] Komdeur, J., Oorebeek, M., van Overveld, T. & Cuthill, I. C. Mutual ornamentation, age, and reproductive performance in the European starling. *Behav. Ecol.* **16**, 805–817 (2005).

[81] Delhey, K. & Kempenaers, B. Age differences in blue tit *Parus caeruleus* plumage colour: within-individual changes or colour-biased survival? *J. Avian Biol.* **37**, 339–348 (2006).

[82] Hidalgo-Garcia, S. The carotenoid-based plumage coloration of adult blue tits *Cyanistes caeruleus* correlates with the health status of their brood. *Ibis* **148**, 727–734 (2006).

[83] Doutrelant, C. et al. Female coloration indicates female reproductive capacity in blue tits. *J. Evol. Biol.* **21**, 226–233 (2008).

[84] Doutrelant, C., Grégoire, A., Midamegbe, A., Lambrechts, M. & Perret, P. Female plumage coloration is sensitive to the cost of reproduction. An experiment in blue tits. *J. Anim. Ecol.* **81**, 87–96 (2012).

[85] Henderson, L. J., Heidinger, B. J., Evans, N. P. & Arnold, K. E. Ultraviolet crown coloration in female blue tits predicts reproductive success and baseline corticosterone. *Behav. Ecol*. **24**, 1299–1305 (2013).

[86] Limbourg, T., Mateman, A. C. & Lessells, C. M. Parental care and UV coloration in blue tits: opposite correlations in males and females between provisioning rate and mate's coloration. *J. Avian Biol.* **44**, 017–026. (2013).

[87] Lucass, C., Iserbyt, A., Eens, M. & Müller, W. Structural (UV) and carotenoid-based plumage coloration - signals for parental investment? *Ecol. Evol.* **6**, 3269–3279 (2016).

[88] Hõrak, P., Ots, I., Vellau, H., Spottiswoode, C. & Møller, A. P. Carotenoid-based plumage coloration reflects hemoparasite infection and local survival in breeding great tits. *Oecologia* **126**, 166–173 (2001).

[89] Ferns, P. N. & Hinsley, S. A. Immaculate tits: head plumage pattern as an indicator of quality in birds. *Anim. Behav*. **67**, 261–272 (2004).

[90] Mänd, R., Tilgar, V. & Møller, A. P. Negative relationship between plumage colour and breeding output in female great tits, *Parus major*. *Evol. Ecol. Res*. **7**, 1013−1023 (2005).

[91] Remeš, V., Matysioková, B. & Klejdus, B. Egg yolk antioxidant deposition as a function of parental ornamentation, age, and environment in great tits *Parus major*. *J. Avian Biol.* **42**, 387–396 (2011).

[92] Kilgas, P. et al. Variation in assemblages of feather bacteria in relation to plumage color in female great tits. *Condor* **114**, 606–611 (2012).

[93] Pickett, S. R. A., Weber, S. B., McGraw, K. J., Norris, K. J. & Evans, M. R. Environmental and parental influences on offspring health and growth in great tits (*Parus major*). *PLoS One* **8**, e69695 (2013).

[94] Remeš, V. & Matysioková, B. More ornamented females produce higher-quality offspring in a socially monogamous bird: an experimental study in the great tit (*Parus major*). *Front. Zool.* **10**, 14 (2013).

[95] Losdat, S. et al. Effects of an early-life paraquat exposure on adult resistance to oxidative stress, plumage colour and sperm performance in a wild bird. *J. Anim. Ecol*. **87**, 1137–1148 (2018).

[96] Surmacki, A., Stępniewski, J. & Stępniewska, M. Juvenile sexual dimorphism, dichromatism and condition-dependent signaling in a bird species with early pair bonds. *J. Ornithol*. **156**, 65–73 (2015).

[97] van Dijk, R. E., Robles, R., Groothuis, T. G. G. , de Vries, B. & Eising, C. M. Reproductive effort of both male and female bar-throated *Apalis Apalis thoracica* is predicted by ornamentation of self and mate. *Ibis* **157**, 731–742 (2015).

[98] Bitton, P-P., Dawson, R. D. & Ochs, C. L. Plumage characteristics, reproductive investment and assortative mating in tree swallows *Tachycineta bicolor*. *Behav. Ecol. Sociobiol.* **62**, 1543–1550 (2008).

[99] Bentz, A. B. & Siefferman, L. Age-dependent relationships between coloration and reproduction in a species exhibiting delayed plumage maturation in females. *J. Avian Biol.* **44**, 080–088 (2013).

[100] Beck, M. L., Hopkins, W. A. & Hawley, D. M. Relationships among plumage coloration, blood selenium concentrations and immune responses of adult and nestling tree swallows. *J. Exp. Biol.* **218**, 3415–3424 (2015).

[101] Griebel, I. A. & Dawson, R. D. Predictors of nestling survival during harsh weather events in an aerial insectivore, the tree swallow (*Tachycineta bicolor*). *Can. J. Zool.* **97**, 81–90 (2019).

[102] Møller, A. P. Female choice selects for male sexual tail ornaments in the monogamous swallow. *Nature* **332**, 640–642 (1988).

[103] Møller, A. P. Sexual selection in the monogamous barn swallow (*Hirundo rustica*). II. Mechanisms of sexual selection. *J. Evol. Biol.* **5**, 603–624 (1992).

[104] Møller, A. P. Sexual selection in the barn swallow *Hirundo rustica*. III. Female tail ornaments. *Evolution* **47**, 417–431 (1993).

[105] Cuervo, J. J., Møller, A. P. & de Lope, F. Experimental manipulation of tail length in female barn swallows (*Hirundo rustica*) affects their future reproductive success. *Behav. Ecol*. **14**, 451–456 (2003).

[106] Safran, R. J. & McGraw, K. J. Plumage coloration, not length or symmetry of tail-streamers, is a sexually selected trait in north American barn swallows. *Behav. Ecol*. **15**, 455–461 (2004).

[107] Vortman, Y., Lotem, A., Dor, R., Lovette, I. J. & Safran, R. J. The sexual signals of the East-Mediterranean barn swallow: a different swallow tale. *Behav. Ecol*. **22**, 1344–1352 (2011).

[108] Saino, N. et al. Viability is associated with melanin-based coloration in the barn swallow (*Hirundo rustica*). *PloS One* **8**, e60426 (2013).

[109] Hasegawa, M., Arai, E., Watanabe, M. & Nakamura, M. Colourful males hold high quality territories but exhibit reduced paternal care in barn swallows. *Behaviour* **151**, 591–612 (2014).

[110] Hasegawa, M., Arai, E., Ito, S. & Wakamatsu, K. High brood patch temperature of less colourful, less pheomelanic female barn swallows *Hirundo rustica*. *Ibis* **158**, 808–820 (2016).

[111] Liu, Y., Scordato, E. S. C., Safran, R. & Evans M. Ventral colour, not tail streamer length, is associated with seasonal reproductive performance in a Chinese population of barn swallows (*Hirundo rustica gutturalis*). *J. Ornithol.* **159**, 675–685 (2018).

[112] Fitzpatrick, S. & Price, P. Magpies' tails: damage as an indicator of quality. *Behav. Ecol. Sociobiol.* **40**, 209–212 (1997).

[113] Blanco, G. & de la Puente, J. Multiple elements of the black-billed magpie's tail correlate with variable honest information on quality in different age/sex classes. *Anim. Behav*. **63**, 217–225 (2002).

[114] Blanco, G. & Fargallo, J. A. Wing whiteness as an indicator of age, immunocompetence, and testis size in the Eurasian black-billed magpie (*Pica pica*). *Auk* **130**, 399–407 (2013).

[115] Regosin, J. V. & Pruett-Jones, S. Sexual selection and tail-length dimorphism in scissor-tailed flycatchers. *Auk* **118**, 167–175 (2001).

[116] McGraw, K. J., Massaro, M., Rivers, T. J. & Mattern, T. Annual, sexual, size- and condition-related variation in the colour and fluorescent pigment content of yellow crest-feathers in Snares penguins (*Eudyptes robustus*). *Emu* **109**, 93–99 (2009).

[117] Massaro, M., Davis, L. S. & Darby, J. T. Carotenoid-derived ornaments reflect parental quality in male and female yellow-eyed penguins (*Megadyptes antipodes*). *Behav. Ecol. Sociobiol.* **55**, 169–175 (2003).

[118] Dobson, F. S., Couchoux, C. & Jouventin, P. Sexual selection on a coloured ornament in king penguins. *Ethology* **117**, 872–879 (2011).

[119] Keddar, I., Couchoux, C., Jouventin, P. & Dobson, F. S. Variation of mutual colour ornaments of king penguins in response to winter resource availability. *Behaviour* **152**, 1679–1700 (2015).

[120] Viblanc, V. A. et al. Mutually honest? Physiological 'qualities' signalled by colour ornaments in monomorphic king penguins. *Biol. J. Linn. Soc*. **118**, 200–214 (2016).

[121] Krebs, E., Hunte, W. & Green, D. Plume variation, breeding performance and extra-pair copulations in the cattle egret. *Behaviour* **141**, 479–499 (2004).

[122] Daunt, F., Monaghan, P., Wanless, S. & Harris, M. P. Sexual ornament size and breeding performance in female and male European shags *Phalacrocorax aristotelis*. *Ibis* **145**, 54–60 (2003).

[123] Childress, R. B. & Bennun, L. A. Sexual character intensity and its relationship to breeding timing, fecundity and mate choice in the great cormorant *Phalacrocorax carbo lucidus*. *J. Avian Biol*. **33**, 23–30 (2002).

[124] Montoya, B., Valverde, M., Rojas, E. & Torres, R. Oxidative stress during courtship affects male and female reproductive effort differentially in a wild bird with biparental care. *J. Exp. Biol*. **219**, 3915–3926 (2016).

[125] Amat, J. A. et al. Dynamic signalling using cosmetics may explain the reversed sexual dichromatism in the monogamous greater flamingo. *Behav. Ecol. Sociobiol*. **72**, 135 (2018).

[126] Amat, J. A. Does the expression of a male plumage trait in female Kentish plovers (*Charadrius alexandrinus*) signal individual quality? *J. Ornithol*. **146**, 287–290 (2005).

[127] Hanssen, S. A. et al. Individual quality and reproductive effort mirrored in white wing plumage in both sexes of south polar skuas. *Behav. Ecol*. **20**, 961–966 (2009).

[128] Doutrelant, C. Colouration in Atlantic puffins and blacklegged kittiwakes: monochromatism and links to body condition in both sexes. *J. Avian Biol*. **44**, 451–460 (2013).

[129] Jones, I. L., Hunter, F. M. & Fraser, G. Patterns of variation in ornaments of crested auklets *Aethia cristatella*. *J. Avian Biol*. **31**, 119–127 (2000).

[130] Douglas, H. D., Kitaysky, A. S., Kitaiskaia, E. V., Maccormick, A. & Kelly, A. Size of ornament is negatively correlated with baseline corticosterone in males of a socially monogamous colonial seabird. *J. Comp. Physiol. B* **179**, 297 (2009).

[131] Klenova, A. V., Zubakin, V. A. & Zubakina, E. V. Vocal and optical indicators of individual quality in a social seabird, the crested auklet (*Aethia cristatella*). *Ethology* **117**, 356–365 (2011).

[132] Palestis, B. G., Nisbet, I. C. T., Hatch, J.J., Arnold, J. M. & Szczys, P. Tail length and sexual selection in a monogamous, monomorphic species, the roseate tern *Sterna dougallii*. *J. Ornithol*. **153**, 1153–1163 (2012).

[133] Velando, A., Lessells, C. M. & Márquez, J. C. The function of female and male ornaments in the Inca tern: evidence for links between ornament expression and both adult condition and reproductive performance. *J. Avian Biol*. **32**, 311–318 (2001).

[134] Sepp, T. et al. A small badge of longevity: opposing survival selection on the size of white and black wing markings. *J. Avian Biol*. **48**, 570–580 (2017).

[135] Pardal, S., Alves, J. A., Mota, P. G. & Ramos, J. A. Dressed to impress: breeding plumage as a reliable signal of innate immunity. *J. Avian Biol*. **49**, e01579 (2018).

[136] Piersma, T. et al. Breeding plumage honestly signals likelihood of tapeworm infestation in females of a long-distance migrating shorebird, the bar-tailed godwit. *Zoology* **104**, 41–48 (2001).

[137] Blizard, M. & Pruett-Jones, S. Plumage pattern dimorphism in a shorebird exhibiting sex-role reversal (*Actitis macularius*). *Auk* **134**, 363–376 (2017).

[138] Veit, A. C. & Jones, I. L. Function of tail streamers of red-tailed tropicbirds (*Phaethon rubricauda*) as inferred from patterns of variation. *Auk* **120**, 1033–1043 (2003).

[139] Mougeot, F., Irvine, J. R., Seivwright, L., Redpath, S. M. & Piertney, S. Testosterone, immunocompetence, and honest sexual signaling in male red grouse. *Behav. Ecol.* **15**, 930–937 (2004).

[140] Mougeot, F., Redpath, S. M. & Leckie, F. Ultra-violet reflectance of male and female red grouse, *Lagopus lagopus scoticus*, sexual ornaments reflect

nematode parasite intensity. *J. Avian Biol*. **36**, 203–209 (2005).

[141] Martínez-Padilla, J. et al. Condition- and parasite-dependent expression of a male-like trait in a female bird. *Biol. Lett.* **7**, 364–367 (2011).

[142] Vergara, P., Martínez-Padilla, J., Redpath, S. M. & Mougeot, F. The ornament-condition relationship varies with parasite abundance at population level in a female bird. *Naturwissenschaften* **98**, 897–902 (2011).

[143] Vergara, P., Redpath, S. M., Martínez-Padilla, J. & Mougeot, F. Environmental conditions influence red grouse ornamentation at a population level. *Biol. J. Linn. Soc.* **107**, 788–798 (2012).

[144] Legagneux, P., Théry, M., Guillemain, M., Gomez, D. & Bretagnolle, V. Condition dependence of iridescent wing flash-marks in two species of dabbling ducks. *Behav. Process.* **83**, 324–330 (2010).

[145] Butler, M. W. & McGraw, K. J. Past or present? Relative contributions of developmental and adult conditions to adult immune function and coloration in mallard ducks (*Anas platyrhynchos*). *J. Comp. Physiol. B* **181**, 551–563 (2011).

[146] Hanssen, S. A., Folstad, I. & Erikstad, K. E. White plumage reflects individual quality in female eiders. *Anim. Behav*. **71**, 337–343 (2006).

[147] Hanssen, S. A., Hasselquist, D., Folstad, I. & Erikstad, K. E. A label of health: a previous immune challenge is reflected in the expression of a female plumage trait. *Biol. Lett.* **4**, 379–381 (2008).

[148] Lehikoinen, A., Jaatinen, K. & Öst, M. Do female ornaments indicate quality in eider ducks? *Biol. Lett.* **6**, 225–228 (2010).

[149] Gladbach, A., Gladbach, D. J., Kempenaers, B. & Quillfeldt, P. Female-specific colouration, carotenoids and reproductive investment in a dichromatic species, the upland goose *Chloephaga picta leucoptera*. *Behav. Ecol. Sociobiol.* **64**, 1779–1789 (2010).

[150] Kraaijeveld, K., Gregurke, J., Hall, C., Komdeur, J. & Mulder, R. A. Mutual ornamentation, sexual selection, and social dominance in the black swan. *Behav. Ecol*. **15**, 380–389 (2004).
