# Supplementary material for: Ornaments are equally informative in male and female birds
Source: Nat Commun. 2022 Oct 7;13:5917. doi: 10.1038/s41467-022-33548-7 (PMC9546859; doi:10.1038/s41467-022-33548-7)
Supplement: Supplementary file 3 — Description of Additional Supplementary Files [file 41467_2022_33548_MOESM3_ESM.pdf]

Description of Additional Supplementary Files

File Name: Supplementary Data 1
Description: PRISMAEcoEvo checklist.

File Name: Supplementary Data 2
Description: : Raw data from which effect sizes were calculated. These include statistics reported in the compiled studies, calculated from data extracted from figures, or provided by the authors.

File Name: Supplementary Data 3
Description: Full dataset of effect sizes used in the meta-analytic models presented in this paper.

File Name: Supplementary Data 4
Description: File containing 50 phylogenetic trees for all bird species included in this paper. All trees were downloaded from https://birdtree.org using Ericson backbone phylogenies.

File Name: Supplementary Code 1
Description: R Code to reproduce the analyses presented in this study.
