# Supplementary material for: Ornaments are equally informative in male and female birds
Source: Nat Commun. 2022 Oct 7;13:5917. doi: 10.1038/s41467-022-33548-7 (PMC9546859; doi:10.1038/s41467-022-33548-7)
Supplement: Supplementary file 9 — Reporting Summary [file 41467_2022_33548_MOESM9_ESM.pdf]

nature portfolio

Corresponding author(s): Sergio Nolazco

Last updated by author(s): 2022/09/18

Reporting Summary

Nature Portfolio wishes to improve the reproducibility of the work that we publish. This form provides structure for consistency and transparency in reporting. For further information on Nature Portfolio policies, see our Editorial Policies and the Editorial Policy Checklist.

Statistics

For all statistical analyses, confirm that the following items are present in the figure legend, table legend, main text, or Methods section.

n/a	Confirmed	
☐	☒	The exact sample size (*n*) for each experimental group/condition, given as a discrete number and unit of measurement
☒	☐	A statement on whether measurements were taken from distinct samples or whether the same sample was measured repeatedly
☐	☒	The statistical test(s) used AND whether they are one- or two-sided<br>*Only common tests should be described solely by name; describe more complex techniques in the Methods section.*
☐	☒	A description of all covariates tested
☒	☐	A description of any assumptions or corrections, such as tests of normality and adjustment for multiple comparisons
☐	☒	A full description of the statistical parameters including central tendency (e.g. means) or other basic estimates (e.g. regression coefficient) AND variation (e.g. standard deviation) or associated estimates of uncertainty (e.g. confidence intervals)
☐	☒	For null hypothesis testing, the test statistic (e.g. *F*, *t*, *r*) with confidence intervals, effect sizes, degrees of freedom and *P* value noted<br>*Give P values as exact values whenever suitable.*
☐	☒	For Bayesian analysis, information on the choice of priors and Markov chain Monte Carlo settings
☐	☒	For hierarchical and complex designs, identification of the appropriate level for tests and full reporting of outcomes
☐	☒	Estimates of effect sizes (e.g. Cohen's *d*, Pearson's *r*), indicating how they were calculated

Our web collection on statistics for biologists contains articles on many of the points above.

Software and code

Policy information about availability of computer code

Data collection	Web of Science, RAYYAN, WebPlotDigitizer (version 4.5), https://birdtree.org/ (phylogenetic trees)
Data analysis	R statistical software (version 3.6.3). R packages: BSDA (version 1.2.0), metafor (version 2.1-0), MCMCglmm (version 2.29). R code for reproducing the results in this study is available as Supplementary Material.

For manuscripts utilizing custom algorithms or software that are central to the research but not yet described in published literature, software must be made available to editors and reviewers. We strongly encourage code deposition in a community repository (e.g. GitHub). See the Nature Portfolio guidelines for submitting code & software for further information.

Data

Policy information about availability of data

All manuscripts must include a data availability statement. This statement should provide the following information, where applicable:

- Accession codes, unique identifiers, or web links for publicly available datasets
- A description of any restrictions on data availability
- For clinical datasets or third party data, please ensure that the statement adheres to our policy

Source data are provided with this paper. A complete raw data file, along with calculated effect sizes (Supplementary Data 2), the prepared dataset (Supplementary Data 3) and the subset of phylogenetic trees (Supplementary Data 4) used to run the analyses, and a list of all references from which the data were extracted

(Supplementary Information) are also provided. Phylogenetic trees can be downloaded directly from https://birdtree.org and species nomenclature used in this paper is available from https://birdsoftheworld.org (accessed on April 2020).

Human research participants

Policy information about studies involving human research participants and Sex and Gender in Research.

Reporting on sex and gender	*Use the terms sex (biological attribute) and gender (shaped by social and cultural circumstances) carefully in order to avoid confusing both terms. Indicate if findings apply to only one sex or gender; describe whether sex and gender were considered in study design whether sex and/or gender was determined based on self-reporting or assigned and methods used. Provide in the source data disaggregated sex and gender data where this information has been collected, and consent has been obtained for sharing of individual-level data; provide overall numbers in this Reporting Summary. Please state if this information has not been collected. Report sex- and gender-based analyses where performed, justify reasons for lack of sex- and gender-based analysis.*
Population characteristics	*Describe the covariate-relevant population characteristics of the human research participants (e.g. age, genotypic information, past and current diagnosis and treatment categories). If you filled out the behavioural & social sciences study design questions and have nothing to add here, write "See above."*
Recruitment	*Describe how participants were recruited. Outline any potential self-selection bias or other biases that may be present and how these are likely to impact results.*
Ethics oversight	*Identify the organization(s) that approved the study protocol.*

Note that full information on the approval of the study protocol must also be provided in the manuscript.

Field-specific reporting

Please select the one below that is the best fit for your research. If you are not sure, read the appropriate sections before making your selection.

☐ Life sciences ☐ Behavioural & social sciences ☒ Ecological, evolutionary & environmental sciences

For a reference copy of the document with all sections, see nature.com/documents/nr-reporting-summary-flat.pdf

Ecological, evolutionary & environmental sciences study design

All studies must disclose on these points even when the disclosure is negative.

Study description	This study is a systematic quantitative review investigating the associations between biological ornaments and indicators of body condition and fitness across mutually ornamented bird species. Data was obtained from peer-reviewed studies. These data were analysed using a phylogenetically controlled bivariate meta-analysis, modelling female and male effects as response variables simultaneously. This approach was selected over univariate meta-analysis as it is superior for dealing with paired data. It provides more precise estimations and reduction in bias because it takes into account correlations between female-male paired effect sizes (i.e., within study dependencies) and at the same time has the advantage of allowing missing data (i.e., observations missing data for one dependent variable). Species identity was incorporated as random effect to account for multiple effects derived from the same species. This random effect roughly explained the same amount of variance as study identity because in most cases there was only one study per species, therefore, only species identity was accounted for as random effect. To control for phylogenetic relatedness, its effects were estimated by means of a phylogenetic correlation matrix. To correct for phylogenetic uncertainty, 50 phylogenetic trees were used and incorporated this uncertainty into posterior distributions. The models were run for 11000 iterations, with a thin of 100 and a burn-in of 1000, resulting in posterior samples of 5000. Seven main models were considered: (1) meta-analytical mean model with sex as a moderator, (2) model with sex and type of ornament as moderators and the interactions between them, (3) model with sex and type of association (condition/fitness) as moderators and their interaction, (4) model with sex and subcategories of indicators of condition as moderators and their interactions, (5) model with sex and subcategories of indicators of fitness as moderators and their interactions, (6) model with sex and degree of ornament sexual dimorphism as moderators and their interaction for condition data only, and (7) model with same structure as the previous one but for fitness data only. All these models included publication year (mean centred to zero) as an additional moderator to control for time-lag bias (i.e., when those studies with larger or significant effects are published quicker than those with smaller or non-significant effects, which translates into a decline over time).
Research sample	The research sample comprises 64 mutually ornamented bird species around the world. The analysis was restricted to birds because it is the most well-studied taxa in the topic. This study was also limited to mutually ornamented species to allow paired data analyses between female and male homologous traits. Our full dataset includes 981 effect sizes Zr (females = 510, males = 471). All these data was extracted from 150 peer-reviewed papers published between 1996 and 2019. Effect sizes represent associations between ornamentation and indicators of body condition and fitness, and were calculated from statistics or raw data extracted from the main text of the studies, supplementary materials, figures, or directly provided by the authors. Indicators of condition were classified into six categories: (1) body condition: mainly measurements of body mass adjusted by structural body size and others associated with the physical condition of individuals; (2) body size: structural size (measurements of tarsus, wing, beak, keel, and tail alone or in combination) and mass; (3) immunity: indicators of constitutive immunity, immune challenges and responses; (4) stress: indicators of baseline physiological stress, stress challenges, and capacity to cope with oxidative stress; (5) environment: climatic conditions and

resources; and (6) parasites: incidence and abundance of parasites. Indicators of fitness were classified into five categories: (1) reproductive success: mating success and offspring production; (2) offspring quality or condition: measurements of egg quality, offspring body condition, immunity, parasites, and other indicators of physical condition; (3) parental quality: provisioning during incubation, and offspring feeding and defence; (4) timing of breeding: measured directly or as arrival time to breeding grounds; and (5) survival. Ornaments involved plumage (n = 862), bill (n = 61), eye (n = 4), bare skin, parts such as feet, gular skin, orbital ring, wattle, comb, and gape (n = 52), and combinations of these traits (n = 2). Among these ornaments there were carotenoid-based ones (n = 285), melanin (n = 188), structural (n = 148), morphological (n = 129), unpigmented (n = 118), and combinations of these or other rare pigments such as spheniscin (n = 113). Our dataset also include effect sizes (Cohen's d) on sexual dimorphism for the ornamental traits investigated, extracted from statistics or raw data from the same publications or directly obtained from the authors. These data were available for only 47 species and 438 effect sizes of our full dataset (64 spp and 981 effect sizes, respectively).

Sampling strategy	No sample size calculation was performed a priori. The search strategy was designed to maximize the amount of compiled studies from which effect sizes were calculated. The combination of a relatively large sample size for meta-analysis (981 effect sizes) and high precision of bivariate random-effects models was intended to ensure accurate results.
Data collection	All data collection, screening process, and variables classification were done by the first author (Sergio Nolazco). The data collector constructed a PRISMA (Preferred Reporting Items for Systematic Reviews and Meta-Analyses) flowchart to report the literature search strategy and study selection process. Data was restricted to peer-reviewed studies from which effect sizes were possible to be calculated.
Timing and spatial scale	The data was extracted after a literature search in the Web of Science (all citation databases) conducted on 12 June 2019. The compiled studies were not restricted to a specific country or region.
Data exclusions	No data (calculated effect sizes) were excluded from the analyses.
Reproducibility	Not applicable because this is not an experimental study. However, all code and data is provided to reproduce the results of this study. The authors have ran all models several times and the results were consistent.
Randomization	Not applicable to this type of study. Data was compiled from literature databases.
Blinding	No blinding was required in this type of study.

Did the study involve field work? ☐ Yes ☒ No

Reporting for specific materials, systems and methods

We require information from authors about some types of materials, experimental systems and methods used in many studies. Here, indicate whether each material, system or method listed is relevant to your study. If you are not sure if a list item applies to your research, read the appropriate section before selecting a response.

Materials & experimental systems

n/a	Involved in the study	
☒	☐	Antibodies
☒	☐	Eukaryotic cell lines
☒	☐	Palaeontology and archaeology
☒	☐	Animals and other organisms
☒	☐	Clinical data
☒	☐	Dual use research of concern

Methods

n/a	Involved in the study	
☒	☐	ChIP-seq
☒	☐	Flow cytometry
☒	☐	MRI-based neuroimaging
